# Strange Metallicity and Magnetic Order in the CoNi(Cr/V) Medium-Entropy Alloy System

**DOI:** 10.3390/ma16031044

**Published:** 2023-01-24

**Authors:** Faisal Mustafa, Mehmet Egilmez, Wael Abuzaid, Sami El-Khatib, Tahir Nawaz, Shahbaz Ahmad, Serhat Alagoz

**Affiliations:** 1 Department of Physics, American University of Sharjah, Sharjah 26666, United Arab Emirates; 2 Materials Science and Engineering Program, American University of Sharjah, Sharjah 26666, United Arab Emirates; 3 Department of Mechanical Engineering, American University of Sharjah, Sharjah 26666, United Arab Emirates; 4 Department of Physics, University of Alberta, Edmonton, AB T6G 2E1, Canada

**Keywords:** medium entropy alloys, CoNiCr, short-range order, strange metallicity

## Abstract

CoNiCr is a prototypical example of topical multi-principle element alloys with superior cryogenic and high-temperature mechanical strength, corrosion, oxidation resistance, and yet-to-be-explored magnetic and electronic functionalities. The remarkable properties of this transition metal ternary system are not only due to atomic radii, electronic configurational mismatch, and atomic volume misfit but are also dependent on the debated magnetically driven chemical short-range order. The current study focuses on the electric and magnetic properties of the single-phase face-centered cubic CoNi(Cr/V) system in which V is introduced to the system at the expense of Cr to fine-tune the volume misfit in the system. All the samples exhibited ultra-small magnetic moments due to the complex magnetic interactions of the constituent elements. The electric transport measurements revealed a strange metallicity evidenced through the observation of the linear temperature dependence of the resistivity. Our findings support the recent theoretical studies on the magnetically driven chemical short-range order of the CoNiCr system.

## 1. Introduction

Materials science is advancing at high speed in the search for new materials and/or combinations of existing materials. Conventional metals and alloys have been pushed toward their saturation limits in terms of their functionalities in recent decades. In general, a single principal element is used (e.g., Fe, Al, Ti alloys, etc.), and various minor constituents are added to enhance and optimize the resulting functionalities. Approximately two decades ago, a new class of materials known as high-entropy alloys (HEA) were introduced by Yeh [1] and Cantor [2,3]. The alloying and composition optimization principles adopted in these systems deviate from conventional alloying technologies through the utilization of multiple principal constituent elements. Early works were focused on the equiatomic compositions, while later efforts have ventured outside of these boundaries in an attempt to further exploit the potential benefits of this class of materials through alloying and composition optimization. Due to the utilization of multiple alloying elements at large percentages, the term Multi-principle Element alloys (MPE) is becoming widely accepted in the literature to describe the various alloys that fall into this category of materials [4,5,6,7,8,9,10,11,12]. From a practical perspective, many of the developed MPE compositions have been shown to exhibit remarkable properties in terms of outstanding cryogenic strength and fracture toughness [13], thermal stability [14], and excellent wear and corrosion resistance [15,16,17]. Unlike traditional high-strength alloys, an increase in both the strength and ductility of the HEAs has been observed under cryogenic conditions [18]. These unique properties highlight these materials’ relevance for many structural and functional applications ranging from electronic and biomedical to the aerospace industries [11,19,20,21]. 

The outstanding properties of the HEA are often linked to the high configurational entropy, sluggish diffusion, and severe distortion of the lattice [1,22]. MPEs are classified into three major categories based on the entropy of mixing (∆S_mix_): (i) high-entropy alloys (HEA) with ∆S_mix_ > 1.61 R, (ii) medium-entropy alloys (MEA) with 0.69R < ∆S_mix_ < 1.61 R, and (iii) low-entropy alloys (LEA) with ∆S_mix_ < 0.69 R, where R is the universal gas constant [3,5]. In terms of the number of constituent elements, most HEA compositions have five or more, while the MEA mostly has three constituent elements [6]. A new horizon of exploration has been opened for researchers with the introduction of the MPE in terms of challenging applications where a combination of physical, chemical, and mechanical functionalities coexists.

Initially, the research focused on the MPE alloys containing five or more elements to obtain the abovementioned properties [3,5]. More recently, it has been observed that the outstanding properties of these MPEs are neither confined to equiatomic composition nor the increasing number of alloying elements (increasing entropy), which was validated after a systematic study on the subsets of the quinary FeNiCoCrMn single-phase alloy [5,23]. Among all the studied compositions, the CoNiCr (MEA) exhibited a significantly higher yield and the highest tensile strength and oxidation and corrosion resistance as compared to other binary, ternary, or quaternary MPEs, including the FeNiCoCrMn system for a large spectrum of temperatures [13,18,24,25,26,27,28,29,30,31,32,33,34,35]. Both from the experimental and theoretical point of view, the CoNiCr MEA is an extremely interesting model system, where the atomic radii mismatch is not so large as to produce an observed solid solution strengthening [29]. Extensive theoretical studies have been carried out to understand the underlying mechanism of the mechanical properties through the calculation of the stacking fault energies (SFEs) of the CoNiCr system [33,36,37]. In this regard, the SFE is a crucial element in understanding the deformation mechanisms of metallic alloys [29,38]. Yet, the atomistic simulations, which consider the CoNiCr system as a random solid solution, have failed to predict the experimental SFE values correctly [28,36,37]. Recently, this discrepancy between the experimental values and atomistic simulations has been shown by several groups by considering the local chemical short-range order (SRO) in the CoNiCr system [18,27,32,34]. Most of these studies, while appropriating the atomistic origin of the observed mechanical functionalities, revealed the importance of the magnetic interactions in the establishment of the short-range order in the CoNiCr system, thus motivating the current study [27,34]. Recently, a link between the resistivity and the SRO in CoNiCr was established [39]. However, there is still no clear consensus on the SRO and its implications; hence, a careful experimental magnetic study on the FCC CoNiCr system is essential to validate the magnetic moment predictions of various models. 

From a different perspective, it has been suggested that the atomic volume misfit has more impact on the phase stabilization and the SFE in the CoNiCr system than the atomic radius mismatch [29,32]. The Cr atom has around 10% more atomic volume than Ni and Co; potentially, many of the observed mechanical properties could be related to this large misfit. The average volume misfit can be further tuned to much higher levels by replacing the Cr with V in the FCC CoNiCr. Indeed, such volume misfit tuning has proven to be very useful. For instance, Luo et al. showed that the functionality of the equiatomic CoNiV MEA went beyond the mechanical performance with a high ultimate tensile strength of 1 GPa at >90% elongation, but it also had a strong potential for utilization in the highly topical hydrogen energy area [30]. Moreover, a recent theoretical work by Kostiuchenko et al. showed that the CoNiV system potentially possessed substantial SRO [32]. Despite the CoNi(Cr/V) system being the subject of a vast number of theoretical studies related to the SRO and the effect of the magnetic interactions on the phase stability and the SFE, very few experimental studies exist on the magnetic and electrical properties of this system [19,40,41]. In this study, an insight into the physical (electric and magnetic) properties of the CoNi(Cr/V) system is provided in the 5–335 K temperature range. Our findings are relevant to recent theoretical studies on the presence of the short-range order in the CoNiCr(V) system. 

## 2. Materials and Methods 

In this study, three MEA compositions were investigated: equiatomic CoNiCr (CNC), CoNi(Cr_0.5_V_0.5_) (CNCV), and equiatomic CoNiV (CNV). All the ingots were fabricated using an arc melting furnace using high-purity elements. A homogenization heat treatment was conducted in an inert Ar atmosphere at 1100 °C for 24 hrs followed by water quenching. Rectangular strips were subsequently machined from the homogenized ingots and cold rolled to an approximately 75% thickness reduction. Finally, each rolled sample was solutionized at 1000 °C for 60 min followed by water quenching to obtain a single phase and fine-grained structure. The microstructures of the finely polished samples were analyzed with a VEGA-3 TESCAN Scanning Electron Microscope (SEM) after etching. The samples were initially ground using 800 to 4000-grit-sized grinding papers and were polished using 5 μm diamond paste. A mixture of 80 mL HCL and 20 mL HNO_3_ was used as an etchant for 30 s (ASTM-E407). Elemental Dispersive X-ray Electron Spectroscopy (EDS) was performed to confirm the chemical composition and elemental distribution. The crystal structure, phase purity, and chemical homogeneity of the studied alloys were analyzed by a Panalytical X’pert^3^ powder X-ray diffractometer (XRD). The magnetic and electrical transport properties were measured using a Cryogenics Ltd. high field measurement system with a vibrating sample magnetometer (VSM) option in the temperature range of 5–335 K. In a typical VSM, the magnetic sample moves (vibrates in a defined axis) in a space where the pick-up coils can detect the induced electric signals proportional to the magnetization of the specimen due to the magnetic flux gradient. Thin strips of material with dimensions of around 4 × 1 × 0.2 mm^3^ were used for the magnetization measurements. The temperature dependence of the resistance was measured using the 4-probe method, which uses a Keithley 2182 A nanovoltmeter and Keithley 2461 source measure unit in delta mode for the highest accuracy. Note that the 4-probe method is essential for the measurement of the resistance of metallic systems, as in these systems the sample would have much smaller resistance than the contacts. In the 4-probe measurement technique, the contact resistance will not influence the measurement. The measurements were carried out on the three samples for each composition, and the reproducibility of the data was validated. 

## 3. Results and Discussion

**Figure 1:**
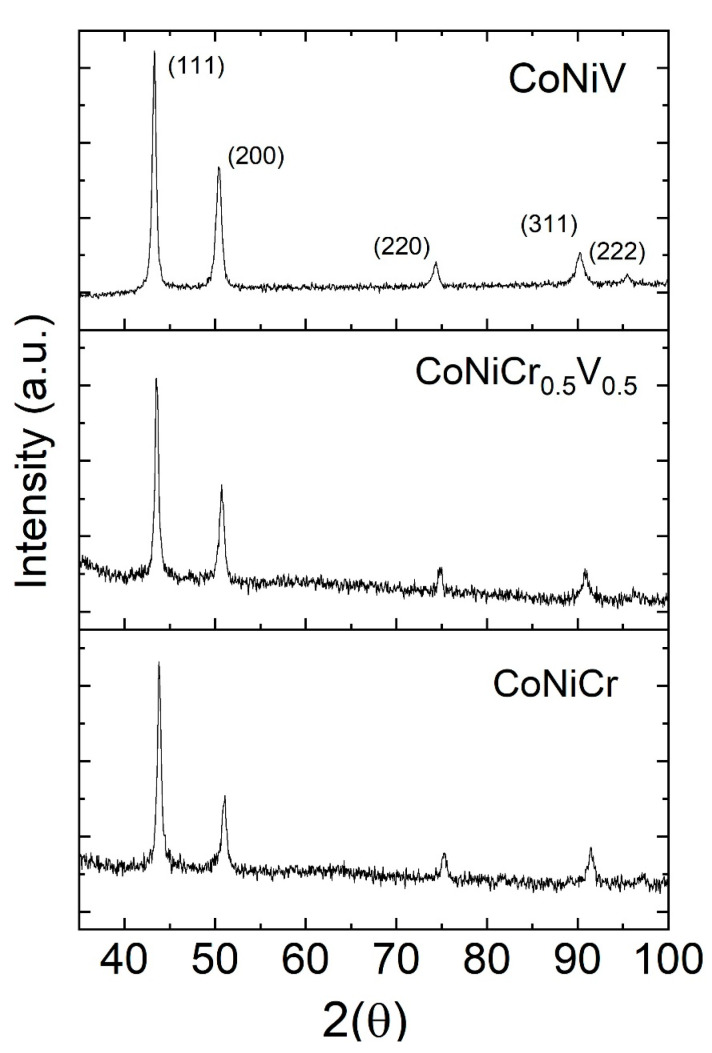
XRD graphs of the CoNiV, CoNiCr_0.5_V_0.5_, and CoNiCr alloys.

The specimens were fully recrystallized after rolling and the adopted solution treatment. X-ray diffraction (XRD) analysis confirmed the presence of the FCC single phase for all three compositions with no traces of any other crystal structure, as shown in Figure 1. The observed FCC peaks were indexed as (111), (200), (220), (311), and (222). The average lattice parameters for the CoNiCr, CoNi(Cr_0.5_V_0.5_), and CoNiV were calculated using the available peaks as 3.576 ± 0.004, 3.598 ± 0.002, and 3.617 ± 0.002 Å, respectively. Vanadium as a larger element in atomic radius than Cr resulted in the monotonic growth of the lattice parameters. 

**Figure 2:**
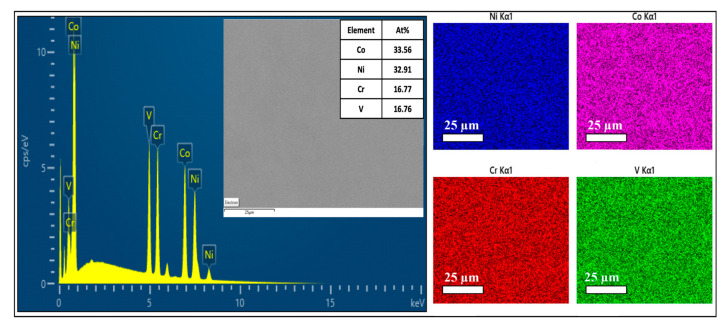
The EDS area mapping of the CoNi(Cr_0.5_V_0.5_) (CNCV) sample.

EDS analyses were performed to determine the elemental composition and distribution on the surface of the specimens. The atomic and weight percentages were verified via EDS analysis, and the results are shown in Table 1. A representative EDS area map graph showing the elemental distribution and elemental atomic percentages along with the corresponding SEM image are shown in Figure 2. The EDS area mapping showed a uniform distribution of the constituent elements, and no agglomeration or intermetallic phases were observed. This observation was valid for all the studied compositions and was in agreement with the XRD analysis presented in Figure 1. 

**Figure 3:**
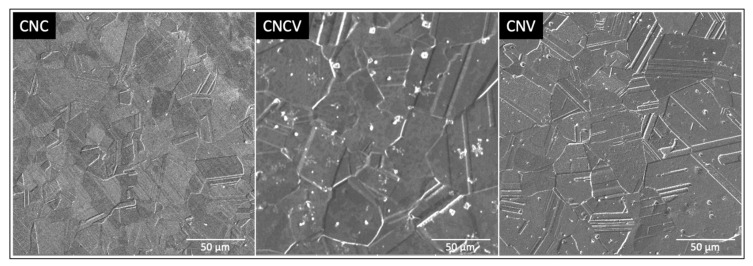
The SEM images of the microstructures of the CNC, CNCV, and CNV alloys.

The grain structure of the alloys was studied using traditional metallurgical procedures on etched polished samples. Following the mechanical rolling and the adopted heat treatments detailed above, the obtained microstructures had grain sizes ranging from 2 to 50 μm, as shown in Figure 3. The CNC and CNV had finer grain structures as compared to the CNCV. In particular, the average grain size for the CNC was around 15–20 μm, and the CNV was around 25–30 μm, while the average grain size for the CNCV system was around 40–50 μm range. Additionally, the density of the annealing twins was higher in the CNV sample. It should be noted here that a detailed discussion of the grain structure in the investigated samples is not within the scope of the current study. However, such variation is expected to affect the resulting mechanical properties in terms of the strength, hardening, and ductility.

**Figure 4:**
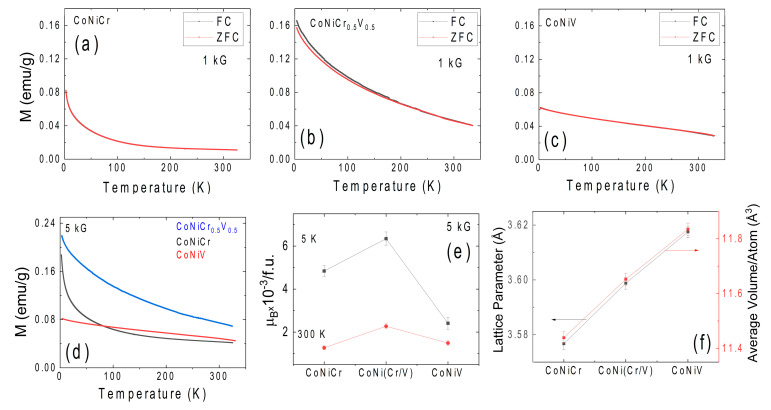
(**a**–**c**) The temperature dependence of the ZFC and FC magnetization of the CNC, CNCV, and CNV measured in a 1kG magnetizing field. (**d**) The temperature dependence of the ZFC magnetization of all the samples measured in 5 kG. (**e**) The compositional dependence of the magnetic moment at 5 K and 300 K. (**f**) The compositional dependence of the Lattice parameter and average atomic volume for all samples.

Figure 4a–c shows the temperature dependence of the field-cooled (FC) and zero-field cooled (ZFC) magnetization measured in a 1 kG applied magnetizing field. No irreversibility in the ZFC and FC curves was observed at any external magnetic fields, which suggests that the magnetic moments were in an equilibrium state. Figure 4d represents the temperature-dependence magnetization in a magnetizing field of 5 kG (ZFC). The temperature dependence of the magnetization for the considered samples resembled what was expected from a paramagnetic material. However, as shown in a subsequent section, all the samples exhibited magnetic hysteresis; hence, they are not the usual molecular field paramagnetic materials. Indeed, Mekata et al. showed that when two magnetic sublattices with slightly different strengths had moments aligned antiparallel, the system had a finite and rather small magnetization. Such a slightly ferromagnetic-like structure is often called a ferrimagnet and could have a temperature dependence of magnetization similar to the ones observed here [42]. Recently, Inoue et al. showed the chemical ordering for the CoNiCr system via atomic probe tomography [43]; Cr tended to occupy the face-centered positions, while the Co/Ni preferred to occupy the cube corner positions in the FCC structure. Such a chemical order supports the formation of two distinct magnetic sublattices, as the Cr moment is known to be negative, while the Co/Ni moments are positive [33]. This aspect is subjected to further discussion below. The magnetizations measured at 5 K revealed magnetic moments of 0.16, 0.21, and 0.08 emu/g for the CNC, CNCV, and CNV, respectively. The calculated magnetic moments per formula unit (f.u.) are shown in Figure 4e for 5 K and 300 K. The calculated magnetic moments were extremely small, as shown in Figure 4e. For instance, the 5 K magnetic moments were measured to be around 0.005, 0.007, and 0.002 μ_B_/f.u. These moments were dramatically lower than the individual moments or constituent elements, which implied the presence of counteracting complex magnetic interactions, as expected in ferrimagnetic or antiferromagnetic states [44].

The magnetism of the CoNiCr system has not been widely investigated in experiments. Sales et al. reported relatively small magnetic moments consistent with our work on the equiatomic CoNiCr system [40,41]. Despite the scarcity of the experimental work, the magnetism of the CoNiCr system and its relation to observed intriguing mechanical properties have been of central interest in many theoretical studies in the last few years. The major motivation for the theoretical studies is to understand the synergy between the strength and the ductility of the CoNiCr system and its relevance to the physical properties, such as the magnetism. The atomic radius misfit between the Co, Ni, and Cr was not large enough to generate significant lattice distortion; hence, from a traditional metallurgical point of view a relatively small solid solution strengthening was expected [29]. In sharp contrast, the CoNiCr system exhibited exceptional solid solution strengthening [13,24]. In an attempt to understand the observed large solid solution strengthening, many atomistic theoretical studies have been carried out on stacking fault energy (SFE) calculations. SFE plays a vital role in understanding the deformation mechanism of a metallic system; hence, atomistic models, which calculate the SFE in close approximation to experimental values, are very useful in understanding the principal deformation mechanisms (i.e., slip, twinning, and phase transformation). Initial attempts to calculate the SFE of the CNC system yielded negative SFE values in the −62 to −24 mJm^−2^ range [33,36,37]. However, the experimentally determined SFE value for the CNC system was positive at 22 mJm^−2^. These initial DFT calculations treated the CNC system as a random solid solution [28]. In an attempt to reduce the discrepancy between the experimental and theoretical SFE values, several research groups have treated the CNC as a chemical short-range order system rather than a quasi-random solid solution [27]. Walsh et al. recently showed that this short-range order (SRO) was driven by the magnetic interactions of constituent elements through spin-polarized DFT calculations [27]. Their calculations revealed a remarkable correlation with the experimental studies on SFE. Despite the success of the theoretical model, direct experimental evidence of the SRO in the CoNiCr system has been intangible, as the atomic mismatch between the Co, Ni, and Cr was quite small. In this regard, we believe that the extremely small magnetic moments reported in this work (see Figure 4) support the findings of the models based on the SRO. Based on the spin-polarized DFT studies of the CNC system, the Ni had a rather passive role (the magnetic moment was suppressed), and the overall magnetic moment of the system was defined by the interactions involving the magnetic Co and the antiferromagnetic Cr [27,28,33,34]. One of the important conclusions of these spin-polarized DFT calculations was the frustration of the magnetic moment of the CNC system, which was in line with our experimental results. More specifically, it was found that the frustration abolished the moments of the Cr atoms bonded to other nearest neighboring Cr atoms. The Cr atoms with fewer Cr neighbors formed a complex spin interaction where frustrated “up” and “down” spin combinations (Cr^↑^ and Cr^↓^) were formed [27]. Through the quantification of the various spin configurations for the first nearest neighbor Cr atom interactions, it was found that magnetically aligned Cr pairs were unlikely in the CNC system, and the systems exhibited an average magnetic moment around 0.28 μ_B_/atom for the random solid solution FCC crystal structure [27]. Such a value of the magnetic moment was much higher than the values reported in Figure 4 d. On the other hand, when higher-order nearest neighbor interactions were considered in the same study, the calculated magnetic moments were very similar to the experimental magnetic moment values reported here. In particular, the Co was ferromagnetically aligned, and its orientation defined the spin ‘up’ state in which the Cr atoms were aligned either in parallel (Cr^↑^) or opposite (Cr^↓^). In the model, a sensible amount of opposite spin Cr was essential to avoid the like-spin Cr pairs for consistency with the experimental results. One of the most noteworthy conclusions of the spin-polarized SRO model suggested by Walsh et al. was predicting the possibility of the spin ordering in the CNC system, hence weak ferromagnetism or ferrimagnetism [27]. According to this prediction formation of the nearest neighbor, Cr^↑^- Cr^↑^, Cr^↑^- Cr^↓^, and Co- Cr^↑^ were less likely than the second nearest neighbor Cr^↓^- Cr^↓^ spin state, and the average magnetic moment at 0 K was calculated to be 0.015 μ_B_/atom [27]. The trend reported in Figure 4d demonstrated the moment of CNC exponentially grew with the decrease in the temperature, and the extrapolation of the data to 0 K would lead to a moment very consistent with the theoretical predictions of the spin-polarized DFT calculations with the SRO. Hence, the magnetization data reported here support the debated chemical short-range order models in the CoNiCr system. Irrespective of the level of the short-range order or models, the magnetic behavior of the CNC system was mostly determined by the Cr-Cr and Co-Cr interactions, and the Ni had a passive role. Although it was not mentioned in the model explicitly, higher-order magnetic interactions are likely to form magnetic sublattices with opposite but unbalanced moments, as expected in a ferrimagnet. Observation of a ferrimagnetic-like ground state here further supports the validity of the models based on the chemical short-range order in the CoNiCr system. 

As mentioned earlier, the atomic radius mismatch between the Co, Ni, and Cr was not large enough to cause the observed traditional solid solution strengthening. Based on the discussion above, the magnetically driven SRO could aid in understanding such phenomena. However, recently, in addition to the role of magnetic interactions, atomic volume misfit along with high shear modulus of the Cr have been suggested to account for the observed solid solution strengthening [29,32]. In this regard, the calculated atomic volumes of Co, Ni, and Cr are 11.12, 10.94, and 12.28 Å^3^. Hence, it is desirable to investigate the effect of the volume misfit on the magnetic properties of the CNC system. To achieve that, the Cr sites were gradually replaced with V, which has a 13.9 Å^3^ atomic volume. Evidence of the average atomic volume expansion can be seen from the measured volume for the studied samples in Figure 3f. Interestingly, the CNCV system exhibited the strongest magnetic response. Vanadium possesses a negative magnetic moment as does Cr; however, the strength of the magnetic moment was much smaller than the Cr moment [44]. Hence, one would expect the frustration of the Vanadium moment would be reduced compared to the Cr only case. Therefore, the net moment of the Co- V^↓^ interaction should increase. However, the reported data here show that a simple explanation of reduced antiferromagnetic coupling between the Co and V did not apply to the CNV alloy, as this alloy had the lowest magnetic moment among the three studied compositions. Indeed, recently, the phase stability and SRO in the CNV system were studied theoretically by Kostiuchenko et al.; it was found that a short-range order existed in this alloy as well, and it involved the strong ordering of Co-V as well as Ni-V as nearest neighbor interactions supplemented by relatively strong repulsion at the second coordination shell [32]. Moreover, they reported that the effect of the spin fluctuations on the pair interactions was small; hence, a small magnetic moment was not unexpected. Nevertheless, more theoretical studies are needed to understand the magnetic trends observed here, as the introduction of Vanadium seemed to activate the magnetic role of Ni in the system. 

**Figure 5:**
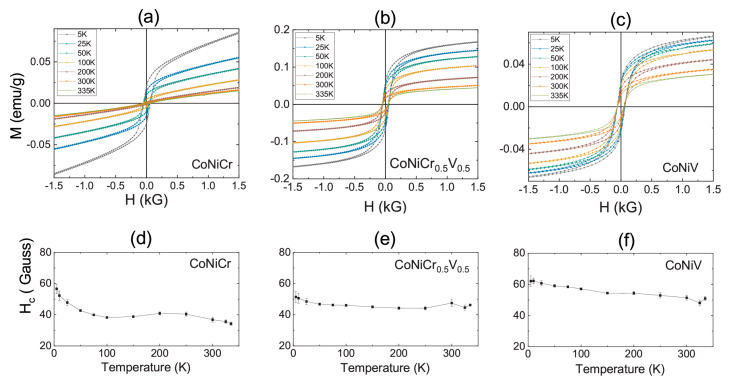
(**a**–**c**) The magnetizing field dependence of the magnetization of the CNC, CNCV, and CNV measured for various temperatures. (**d**–**f**) The temperature dependence of the coercive field of all samples.

**Figure 6:**
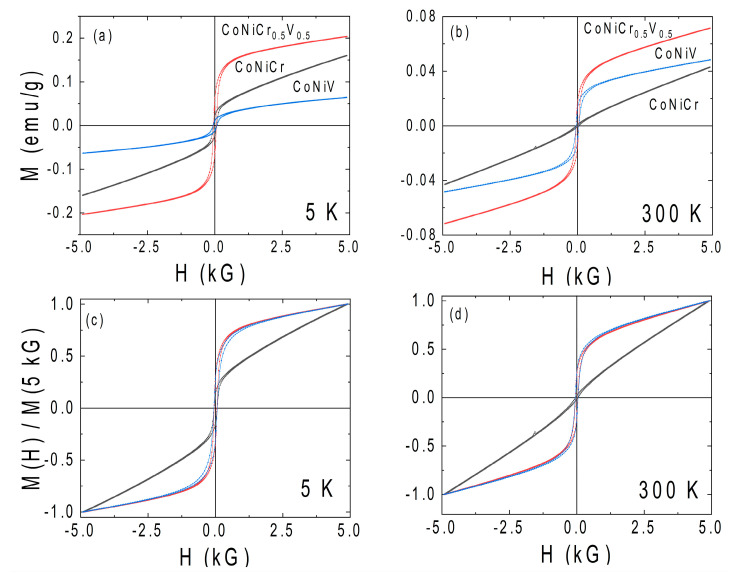
(**a**,**b**) The magnetic moment comparison of all samples for 5 K and 300 K. (**c**,**d**) The field dependence of the magnetization normalized to a magnetization value of 5 kG for the indicated temperatures.

The reported mechanical properties in the CoNiCr(V) system make this group material very interesting for magnetic applications that concern bulk and thin films. In addition to their magnetic moments, one of the most significant factors for the suitableness of this new group of materials in practical applications is its magnetic coercivity. The measured coercive fields (Hc) of all the studied alloys are reported in Figure 5. Apart from the strength of the magnetization, the field dependence of the magnetization of the CNC system was very different to that of the CNCV and CNV. Namely, the field dependence of the magnetization of the CNC system exhibited a very noticeable nonsaturating field dependence up to fields of 90 kG (not shown here). The linear growth of the magnetization with the applied field was consistent with the above discussion on the antiferromagnetic alignment of the moments of Co and Cr [19]. On the other hand, the field dependence of the magnetization of the CNCV and CNV systems showed the signature of magnetic saturation, although they both exhibited a small linear contribution due to the antiferromagnetic coupling between the V and Co and possibly Ni. Figure 5d–f represent the temperature evolution of the coercive field of the three alloys in the 5 K–335 K range. The measured coercive fields were in the range of 30–60 Gauss (2.4–4.8 kA/m), as seen in Figure 5d. Such values of the coercive fields placed the studied alloys into the semihard magnet category. Despite the fact that all the studied alloys exhibited extremely small magnetic moments, the observation of the coercive fields at all temperatures indicated that the magnetic ground state of these alloys was most likely ferrimagnetism, which is consistent with the conclusions drawn from the temperature dependence of the magnetization. In other words, the frustration and antiferromagnetism resulting from Cr and V were strong enough to suppress the overall magnetic moment of the alloy system but still not enough to set the ground state as antiferromagnetic. Consistent with recent theoretical studies on spin-polarized DFT calculations, higher-order magnetic interactions are possibly driving the formation of opposite but unbalanced magnetic sublattices [27]. Note that the ferrimagnetic Curie temperature was not determined in this work due to the setup limitations, which prevented high-temperature measurements. Although all three alloys exhibited a possible ferrimagnetic ground state, the comparison of the field dependence of the magnetization presented in Figure 6a,b gives hints that the magnetism in the CNC system was significantly different than the Vanadium-substituted counterparts. To further highlight the difference, the field dependence of the magnetization of each sample was normalized to the respective magnetization value at 5 kG and plotted, as shown in the insets of Figure 6c,d. When normalized, the field dependence of the magnetization of the CNCV and CNV were almost identical. Such an observation supports the conclusion of reduced frustration in the Vanadium-substituted specimens. 

**Figure 7:**
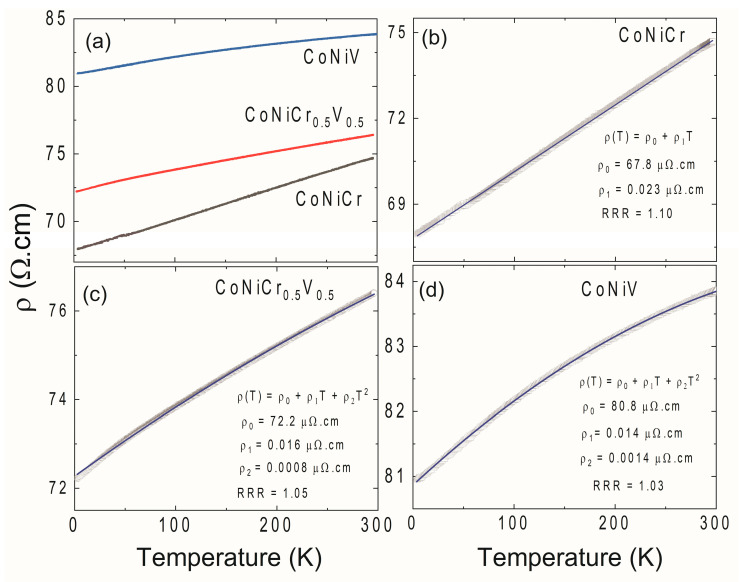
(**a**) The temperature dependence of the resistivity measured in the range of 2–300 K. (**b**–**d**) The power law fits to various models. The solid lines are fits to the expressions in the panel.

In addition to the magnetization, the resistivity of the considered MEA alloys was investigated. The temperature versus resistivity curves of all three compositions in the range of 2–300 K are shown in Figure 7. The resistivity dropped along with the temperature, which was typical metallic behavior. The resistivity of the CoNiCr system can be described by ρ(T) = ρ_0_ + ρ_1_ T, where ρ_0_ is the residual resistivity value, and ρ_1_ is the resistive constant [40,45]. The solid blue line in Figure 7b is a fit to the linear equation, and the fitting parameters are given in the graph. This linear temperature dependence of the resistivity was consistent with recent measurements reported by Sales et al. [40,41]. Linear resistivity is an unusual phenomenon and is often called non-Fermi liquid behavior or strange metallicity [46]. Traditionally such behavior is observed at the proximity of a quantum critical point, where the critical dynamics can lead to an odd time and temperature dependence of correlation functions including the resistivity of the sample [47,48]. Indeed, alternative explanations for the linear temperature dependence of resistivity for simpler systems exist, and they are mostly explained in the limit of dilute metallicity, the low carrier density together with electron–phonon interaction is capable of producing resistive behavior representing strange metallicity without incorporating any strong correlation, electron interaction, or quantum criticality effects [49]. Although the ternary CoNiCr system looks like a simple alloy formed of three rather simple elements, it is an extremely complex system. Sales et al. reported quantum critical behavior in the asymptotic limit of large disorder in the CoNiCr system through detailed analysis of the specific heat and magnetization [41]. In particular, the divergence of the magnetic Gruneisen parameter demonstrated the quantum criticality of this system [41]. Hence, the linear temperature dependence of the resistivity is justified. This linear temperature dependence of resistivity persisted in the Vanadium-substituted samples. However, we added the standard electron–electron scattering term (T^2^ dependence) to better fit the results in these samples. The blue solid lines in Figure 7c,d were fit to a ρ(T) = ρ_0_ + ρ_1_ T + ρ_2_ T^2^ power law expression [50]. The presence of the electron–electron scattering term (similar to standard metallic systems) in the Vanadium-substituted samples postulated a deviation from quantum criticality in CNCV and CNV systems. One additional trend in the data presented in Figure 7 was the increase in the ρ_0_ with an increase in the V concentration. In general, ρ_0_ represents the residual resistivity linked to the imperfections and impurities in the sample. The increase in the volume misfit as shown in Figure 4 f can also increase the residual resistivity. One additional measure of the ‘cleanliness’ of the samples in regard to defect and impurity levels was simply the residual resistivity ratios defined as RRR = ρ(300 K)/ρ(2 K). The RRR in the investigated samples monotonically decreased with the Vanadium substitution supporting the interpretation of the increase in residual resistivity with Vanadium substitution. These large residual resistivity values are expected in granular systems (see Figure 3); the mean free paths of charge carriers are long enough to avoid grain boundaries, but their densities are high enough to lead to large residual resistance values. As a final note, any significant magnetoresistance was not observed in any of the samples. Only limited levels of magnetoresistance were measured, which were notably lower than the results reported by Sales et al. [41].

## 4. Summary

The CoNiCr and CoNiV medium entropy alloys are among the most promising candidates for practical applications due to their superior performance under mechanical (i.e., strength, ductility, and fracture toughness), oxidative (e.g., corrosive), and tribological applications. Despite the strong interest from researchers, there have been only a few experimental studies on the magnetic and electric transport properties of the CoNi(Cr/V) system so far. In this work, ternary equiatomic compositions of CoNiCr, CoNi(Cr_0.5_V_0.5_), and CoNiV were prepared using the arc melting method. Homogenized and solution-treated bulk metallic samples were used to investigate the magnetic and electric properties. The considered MEA compositions all exhibited fine grains and FCC single-phase crystal structures, with monotonic growth in the lattice parameter with the V addition due to the atomic volume mismatch between Cr and V. In particular, detailed temperature and magnetic field dependent magnetization measurements were carried out in the 5–335 K range. The measured magnetic moments at 5 K were determined to be around 0.005, 0.007, and 0.002 μB/f.u for the CoNiCr, CoNi(Cr_0.5_V_0.5_), and CoNiV respectively. These moments were dramatically lower than the individual moments or constituent elements, which implied the presence of counteracting complex magnetic interactions as expected in ferrimagnetism. The electric transport measurements revealed strange metallicity through the observation of the linear temperature dependence of the resistivity. This observation was in line with the recently suggested quantum critical behavior in the CoNiCr system. Our findings support the recent theoretical studies on the magnetically driven chemical short-range order in the CoNiCr system. We believe different electronic band configurations confined in the FCC structure cause small moment frustration in the antiferromagnetic matrix form of (Co/Ni) ^↑^ and (Cr/V) ^↓^, and as a result, a ferrimagnetic-like complex state emerges, which cannot be explained with traditional magnetic theory. The magnetic frustration produces the observed small coercivity, the paramagnetic-like susceptibility behavior, and the quantum critical linearity in the resistivity. Careful experimental tools, such as muon spin resonance and/or neutron reflectivity, would shed light on this magnetic SRO driven by a state of frustration.

## Figures and Tables

**Table 1:** Elemental composition of the samples in atomic and weight percentages.

Element	CoNiCr		CoNi(Cr_0.5_V_0.5_)		CoNiV	
	At.%	Wt.%	At.%	Wt.%	At.%	Wt.%
Ni	33.15	34.42 ±0.14	32.91	34.29 ± 0.13	33.34	34.79 ± 0.15
Co	33.45	34.87 ± 0.13	33.56	35.10 ± 0.12	33.28	35.14 ± 0.14
Cr	33.4	30.71 ± 0.10	16.77	15.47 ± 0.07	─	─
V	─	─	16.76	15.14 ± 0.07	33.38	30.07 ± 0.11

## Data Availability

The data that support the findings of this study are available from the corresponding author upon reasonable request.
